# Epidemiology of *Plasmodium knowlesi* malaria in north-east Sabah, Malaysia: family clusters and wide age distribution

**DOI:** 10.1186/1475-2875-11-401

**Published:** 2012-12-05

**Authors:** Bridget E Barber, Timothy William, Prabakaran Dhararaj, Fread Anderios, Matthew J Grigg, Tsin W Yeo, Nicholas M Anstey

**Affiliations:** 1 Global Health Division, Menzies School of Health Research, PO Box 41096, Casuarina 0810,, Northern Territory, Australia; 2 Infectious Diseases Department, Queen Elizabeth Hospital, Karung Berkunci No. 2029, Jalan Penampang, Kota Kinabalu, 88560, Sabah, Malaysia; 3 Sabah Department of Health, Kota Kinabalu, Sabah, Malaysia; 4 Kudat District Hospital, Peti Surat No. 22, 89057, Kudat, Sabah, Malaysia; 5 Sabah Public Health Reference Laboratory, Bukit Padang, Jalan Kolam, 88850, Kota Kinabalu, Sabah, Malaysia; 6 Royal Darwin Hospital, Darwin, NT, Australia

**Keywords:** *Plasmodium knowlesi*, Malaria, Epidemiology

## Abstract

**Background:**

The simian parasite *Plasmodium knowlesi* is a common cause of human malaria in Malaysian Borneo, with a particularly high incidence in Kudat, Sabah. Little is known however about the epidemiology in this substantially deforested region.

**Methods:**

Malaria microscopy records at Kudat District Hospital were retrospectively reviewed from January 2009-November 2011. Demographics, and PCR results if available, were recorded for each positive result. Medical records were reviewed for patients suspected of representing family clusters, and families contacted for further information. Rainfall data were obtained from the Malaysian Meteorological Department.

**Results:**

*“Plasmodium malariae”* mixed or mono-infection was diagnosed by microscopy in 517/653 (79%) patients. Of these, PCR was performed in 445 (86%) and was positive for *P. knowlesi* mono-infection in 339 (76%). Patients with knowlesi malaria demonstrated a wide age distribution (median 33, IQR 20–50, range 0.7-89 years) with *P. knowlesi* predominating in all age groups except those <5 years old, where numbers approximated those of *Plasmodium falciparum* and *Plasmodium vivax*. Two contemporaneous family clusters were identified: a father with two children (aged 10–11 years); and three brothers (aged one-11 years), all with PCR-confirmed knowlesi malaria. Cases of *P. knowlesi* demonstrated significant seasonal variation, and correlated with rainfall in the preceding three to five months.

**Conclusions:**

*Plasmodium knowlesi* is the most common cause of malaria admissions to Kudat District Hospital. The wide age distribution and presence of family clusters suggest that transmission may be occurring close to or inside people’s homes*,* in contrast to previous reports from densely forested areas of Sarawak. These findings have significant implications for malaria control. Prospective studies of risk factors, vectors and transmission dynamics of *P. knowlesi* in Sabah, including potential for human-to-human transmission, are needed.

## Background

In recent decades Malaysia has achieved a dramatic reduction in malaria prevalence as a result of intensive control efforts, particularly indoor residual spraying and distribution of insecticide-treated nets, with cases falling from 60,000 in 1995 to 6,650 in 2010 [1]. Consequently, Malaysia is in the pre-elimination phase of malaria control and aims to be malaria-free by 2020 [2]. Recently however, the zoonotic infection *Plasmodium knowlesi* has emerged as a common and potentially fatal cause of human malaria in Malaysian Borneo [3-7], and presents an increasing threat to malaria control. Predominantly a parasite of the long-tailed and pig-tailed macaques and transmitted by the forest-dwelling *Anopheles leucosphyrus* group of mosquitoes, *P. knowlesi* is now the most common cause of human malaria in several districts throughout Sabah and Sarawak [3,4,8,9]. The highest proportion has been reported at Kudat District Hospital (KDH), on the north-east tip of Sabah, where 87% of patients admitted with malaria in 2009 were infected with *P. knowlesi*[8].

Little is known however about the epidemiology of *P. knowlesi* in this region. Recent studies conducted in the densely forested Kapit District in Sarawak found that *P. knowlesi* primarily infected adults with a recent history of forest exposure, and that clustering of cases did not occur [4,10]. These findings suggest that in this region transmission occurs in forested areas away from people’s homes, and that human-to-human transmission, although demonstrated experimentally [11], does not appear to be occurring. In contrast to Kapit however, Kudat District has undergone significant deforestation and hence represents a different environmental setting. In addition, mosquito vectors differ between the two regions. In Kapit, *Anopheles latens* has been identified as the *P. knowlesi* vector [12], while in Sabah, although the *P. knowlesi* vector is not yet known, recent studies suggest that *Anopheles balabacensis* and *Anopheles donaldi* are the primary vectors of human malaria [13,14]. Transmission dynamics in Kudat may therefore differ from those previously described in Sarawak. The epidemiology of malaria in Kudat from 2009–2011 was therefore investigated.

## Methods

### Study setting

**Figure 1:**
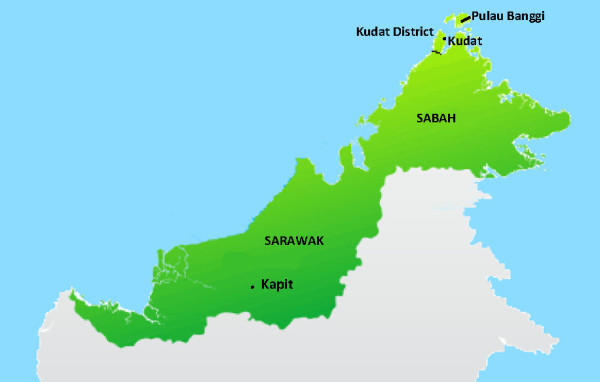
Map of Sarawak and Sabah.

Kudat District (Figure 1) is located on the north-east tip of Borneo and covers an area of 1,287 km^2^, with a population of 83,000 people of predominantly Rungus ethnicity. The district has been substantially deforested, with significant areas of rubber, palm oil and coconut plantations. Located 220 km north of the Equator, it has a tropical climate with no dry season and the maximum rainfall generally corresponds to Sabah’s north-east monsoon season from November to March. Temperatures are fairly constant throughout the year with an average monthly mean of 27°C. Kudat District incorporates the densely forested Pulau Banggi group of islands, the largest of which has an area of 440 km^2^, a population of approximately 20,000 and a main town located 24 km, or one hour by boat, from Kudat. Macaques are numerous throughout Kudat District, including Kudat Town, and are frequently found close to houses.

KDH is the only hospital in the district, and serves as the referral base for five subsector government clinics. All clinics have microscopy facilities and are obliged to refer patients with slides positive for malaria parasites to KDH for admission.

### Laboratory procedures

In this retrospective study blood slides were taken from all febrile patients seen at health clinics, or KDH emergency or outpatient departments, and examined by experienced laboratory microscopists at the clinics or KDH respectively. For clinic patients referred to KDH, slides were repeated and malaria treatment commenced on arrival at KDH. In Sabah, blood slides with parasites resembling *Plasmodium malariae/P. knowlesi* are reported most commonly as “*P. malariae*”*,* but sometimes as “*P. malariae/P. knowlesi*” or *“P. malariae (?P. knowlesi)*”. For simplicity these microscopy reports are all referred to in this paper as “*P. malariae*”. During the study period policy at KDH was to send all these slides to Sabah State Reference Laboratory for PCR confirmation. However, PCR was not routinely performed on the majority of patients diagnosed with other malaria species. At the Sabah State Reference Laboratory *Plasmodium knowlesi* was identified by nested PCR as described elsewhere [10] until June 2011 when a real-time PCR assay was instituted [15]. *Plasmodium falciparum*, *P. vivax*, *P. malariae* and *Plasmodium ovale* were identified using a species-specific real-time PCR assay [16]. Measures taken to minimize contamination in the laboratory included the use of separate workstations for DNA extraction, mastermix preparation, and addition of DNA template; and the use of filter micropipette tips. One negative and one positive control (*P. vivax* or *P. falciparum*) were used during DNA extraction, and a reagent control was used during preparation of the PCR mastermix. *Plasmodium falciparum*, *P. vivax* and *P. malariae* positive controls were sourced from CDC Atlanta (Stephanie Johnston), while the *P. knowlesi* positive control was sourced from the Institute of Medical Research, Kuala Lumpur.

### Retrospective review

Laboratory microscopy records were reviewed for total number of blood slides taken and all blood slides positive for malaria parasites from January 2009 to November 2011 (with results from 2009 briefly reported in part [8]). Date, age, and sex were recorded for all positive results. In addition, PCR results from Sabah State Reference Laboratory were recorded if available. Medical records were reviewed for those patients with *P. knowlesi* suspected of representing family clusters, based on patients with the same family name presenting within a four-week period, and these families were contacted for further information.

Rainfall was recorded using a rain gauge at Kudat Meteorological Station, located one km from the hospital. Monthly rainfall recorded during the study period was obtained from the Malaysian Meteorological Department.

### Statistical analysis

Data were analysed using Stata statistical software, version 10.0 (StataCorp LP, College Station, TX, USA). All analyses were performed on patients with PCR-confirmed *P. knowlesi* mono-infection, and microscopy-diagnosed *P. falciparum* or *P. vivax* mono-infection, due to the small number of PCR assays performed on these latter species. Five patients with microscopy-diagnosed *P. falciparum* but found to have *P. knowlesi* by PCR were excluded from analyses of *P. falciparum* cases. Median ages of patients infected with each of the malaria species were compared using Wilcoxon rank-sum test, and proportions were assessed using the Chi-square test. The Chi-square test was also used to determine inter-annual variation in malaria cases (as a proportion of the total number of slides taken), while seasonality of *P. knowlesi* cases was assessed using Edward’s test. Spearman’s correlation was used to assess the association between rainfall and monthly *P. knowlesi* cases, with cross-correlations analysed to determine the time lag (up to six months) at which the strongest association occurred.

### Ethics statement

The study was approved by the Medical Research Sub-Committee of the Malaysian Ministry of Health and the Health Research Ethics Committee of Menzies School of Health Research, Australia. Ethical approval to contact patients by phone was obtained.

## Results

### Malaria species distribution

From January 2009 to November 2011, 653 (3.4%) patients had slides positive for malaria parasites (Table 1), out of a total of 18,993 slides. “*Plasmodium malariae”* mixed or mono-infection was reported in 517 patients (79%). Of these, PCR was performed in 445 (86%), and was positive for *P. knowlesi* mono-infection in 339 (76%). The remaining 24% included 40 (9%) *P. knowlesi* mixed infections, 23 (5%) *P. vivax* mono-infections, 13 (3%) *P. falciparum* mono-infections, 20 (4%) positive only for *Plasmodium genus*, nine (2%) negative results, and one mixed *P. falciparum*/*P. malariae.* Of the total 445 patients diagnosed by microscopy with* P. malariae *mixed or mono-infection and who had PCR performed, only four (1%) were *P. malariae* by PCR (one *P. falciparum*/*P.malariae*, one *P. malariae*/*P. knowlesi* and two *P. vivax*/*P. malariae*/*P. knowlesi*). Only 35 patients diagnosed by microscopy with *P. falciparum* or *P. vivax* mono-infection had PCR performed. Of these, *P. knowlesi* mono-infection was identified in 5/16 (31%) patients with microscopy-diagnosed *P. falciparum* mono-infection, and mixed *P. knowlesi/P. vivax* infection was identified in 6/19 (32%) patients with microscopy-diagnosed *P. vivax* mono-infection. There were no *P. knowlesi/P. vivax* mixed infections diagnosed by the real-time PCR assay subsequent to June 2011 despite an increase in *P. vivax* mixed and mono-infections diagnosed by microscopy over this time. One additional *P. knowlesi* mono-infection was identified by PCR in a patient with microscopy-diagnosed *P. falciparum/P. vivax* mixed infection, giving a total of 345 patients with PCR-confirmed *P. knowlesi* mono-infection.

**Table 1:** Microscopy and PCR results, January 2009 to November 2011

	**PCR**												
**Microscopy**	**Pf**	**Pv**	**Pk**	**Pv/Pk**	**Pf/Pk**	**Pf/Pv/Pk**	**Pm/Pk**	**Pf/Pm**	**Pv/Pm/Pk**	**P.genus**	**negative**	**Not done**	**Total**
**Pf**	10	0	5	0	0	0	0	0	0	0	1	60	**76**
**Pv**	0	9	0	6	0	0	0	0	0	4	0	34	**53**
**Pm**	3	11	306	18	6	2	1	0	0	18	7	57	**429**
**Pm/Pv**	3	12	10	11	0	0	0	0	1	2	1	7	**47**
**Pf/Pm**	7	0	22	0	0	0	0	1	1	0	1	8	**40**
**Pf/Pv**	1	2	1	1	0	0	0	0	0	0	0	2	**7**
**Pf/Pv/Pm**	0	0	1	0	0	0	0	0	0	0	0	0	**1**
**Total**	**24**	**34**	**345**	**36**	**6**	**2**	**1**	**1**	**2**	**24**	**10**	**168**	**653**

*Blood slides with parasites resembling *P. malariae* were sometimes reported as “Pm/Pk” or “Pm(?Pk)” but for simplicity are all referred to here as “Pm”.Abbreviations: Pf=*P*. *falciparum,* Pv=*P. vivax,* Pm=*P. malariae,* Pk=*P. knowlesi*.

Based on these results, and using the population of the Kudat Division, the estimated incidence of malaria from 2009 to 2011 was 2.6/1,000 people/year, with a minimum incidence of PCR-confirmed *P. knowlesi* mono-infections of 1.4 infections/1,000 people/year.

### Age and sex distribution

**Figure 2:**
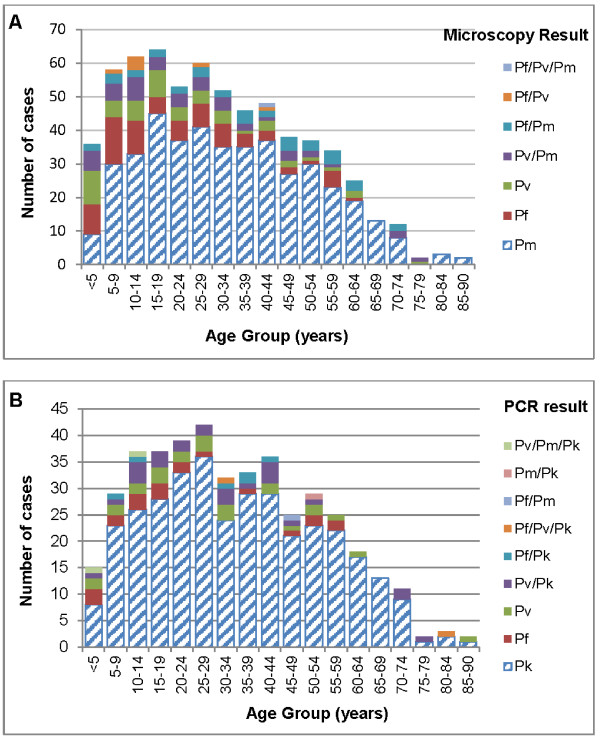
**Age distribution of patients with malaria by microscopy and PCR, January 2009 to November 2011.A**. Age distribution of patients with malaria diagnosed by microscopy; **B**. Age distribution of patients with malaria diagnosed by PCR. Abbreviations: Pf=*P*. *falciparum,* Pv=*P. vivax,* Pm=*P. malariae,* Pk=*P. knowlesi.*

Patients with knowlesi malaria demonstrated a wide age distribution with the species being most common in all age groups except those <5 years old, where numbers approximated those of *P. falciparum* and *P. vivax* (Figure 2). Although the median age of patients with PCR-confirmed *P. knowlesi* (33 years, IQR 20–50 years) was significantly older than patients diagnosed by microscopy with *P. falciparum* (19 years, IQR 9–31 years, p < 0.001) or *P. vivax* (19 years, IQR 7–32 years, p < 0.001), 17% of all PCR-confirmed *P. knowlesi* cases occurred in children <15 years old. Eight children <5 years old had PCR-confirmed knowlesi malaria (with one of these already reported [8]), in addition to two with microscopy-diagnosed “*P. malariae*”. The youngest child with PCR-confirmed knowlesi malaria was eight months old.

Among patients with PCR-confirmed *P. knowlesi* malaria, 252 (73%) were male. This proportion was lower among children (<15 years) than it was among adults (33/57 [58%] *vs* 219/288 [76%], p = 0.005), and highest among adults aged 15–40 years, of whom 133/157 (85%) were male compared to 119/188 (63%) of patients outside this age range (p < 0.001).

Overall the median age of females with PCR-confirmed knowlesi malaria (39 years, IQR 14–53 years) was not significantly different than that of males (32 years, IQR 21.5-49 years, p = 0.770), however among adults with knowlesi malaria, females were significantly older than males (median age 45 years [IQR 30–57 years] *vs* 37 years [IQR 25–52 years], p = 0.004).

### Temporal variation

**Figure 3:**
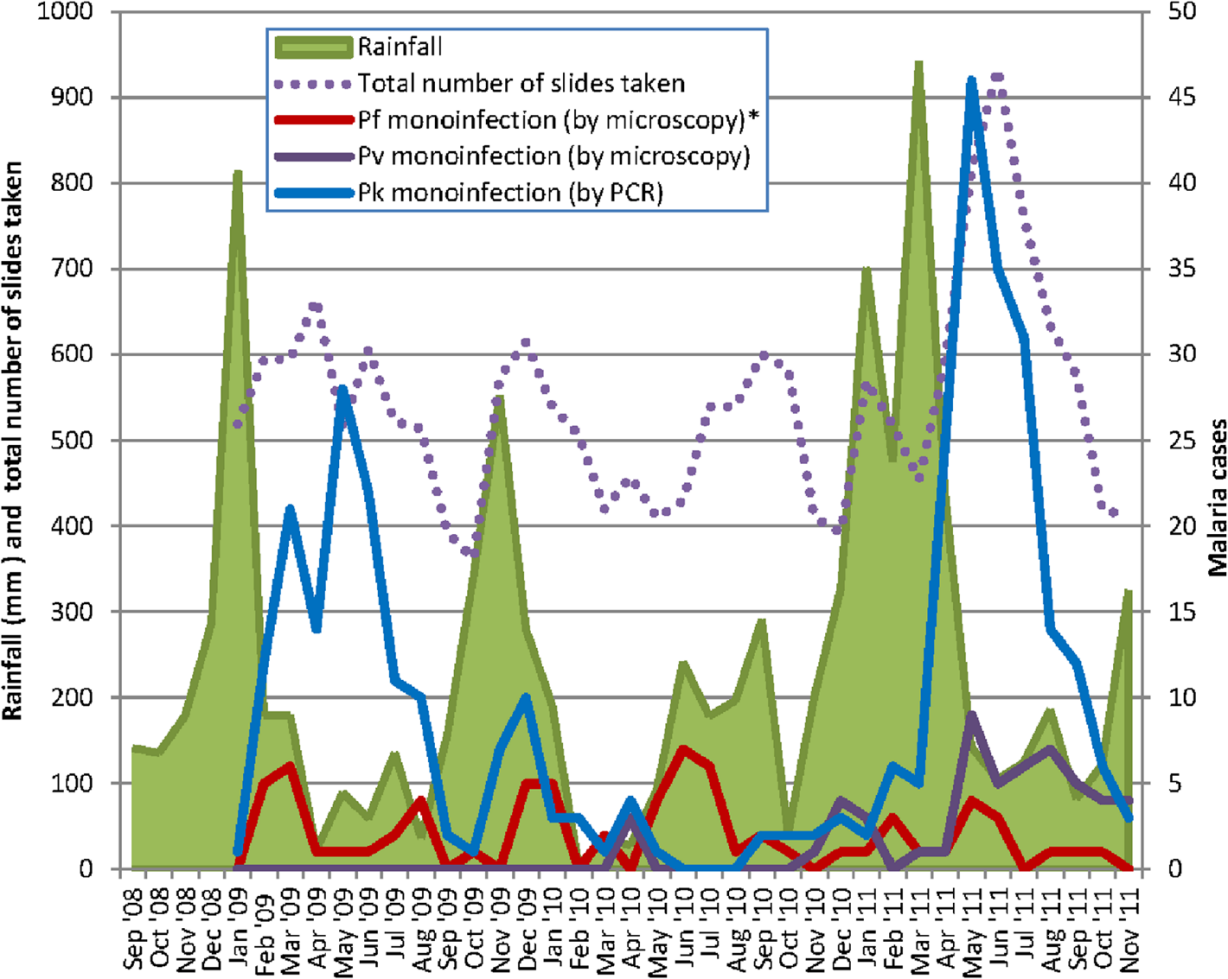
**Monthly rainfall and malaria cases.** *excludes five patients diagnosed by microscopy with *P. falciparum* but found to have *P. knowlesi* by PCR. Abbreviations: Pf=*P*. *falciparum,* Pv=*P. vivax,* Pm=*P. malariae,* Pk=*P. knowlesi.*

The number of patients diagnosed with PCR-confirmed *P. knowlesi* mono-infection as a proportion of the total number of slides taken demonstrated significant inter-annual variation (Figure 3), with 139 (2.1%), 21 (0.4%) and 185 (2.8%) cases diagnosed in 2009, 2010 and 2011 respectively (*χ*^2^ = 107, p < 0.001). Significant seasonality was also demonstrated (p < 0.001), with increased transmission from March to July in 2009 and April to August in 2011. In both years the number of cases peaked in May, with the peak being particularly marked in 2011. Remarkably few cases of knowlesi malaria occurred in 2010.

Rainfall recorded at Kudat meteorological station was strongly correlated with the number of cases of *P. knowlesi* in the proceeding three to five months (Table 2). The number of *P. knowlesi* cases peaked during the fifth month following the rainfall, with a correlation coefficient of 0.50. Total rainfall in 2010 (1,830 mm) was significantly less than in 2009 (2,848 mm) and 2011 (3,643 mm).

**Table 2:** Correlation between monthly rainfall (mm) and number of *P. knowlesi *cases

**Lag time between rainfall and *P. knowlesi *cases (months)**	**Correlation coefficient**	**P value**
0	−0.246	0.156
1	0.122	0.484
2	0.280	0.103
3	**0.469**	**0.005**
4	**0.491**	**0.003**
5	**0.504**	**0.002**
6	0.331	0.060

The number of cases of microscopy-diagnosed *P. falciparum* mono-infections (excluding the five patients found to be *P. knowlesi* by PCR) decreased slightly in the last year of the study period, with 26 (0.4%), 29 (0.5%) and 16 (0.2%) cases diagnosed in 2009, 2010 and 2011 respectively (*χ*^2^ = 6, p = 0.056). In contrast, microscopy-diagnosed *P. vivax* mono-infections increased, with 45 (0.7%) cases in 2011 compared to zero and eight (0.1%) in 2009 and 2010 respectively (*χ*^2^ = 60, p < 0.001).

### Family clusters

Two family clusters of patients with PCR-confirmed *P. knowlesi* monoinfection were identified. The first consisted of a 37 year-old man with his 10 year-old daughter and 11 year-old son, who all presented on the same day with the same duration of illness. The family lived in town, the children attended the local school, and the father worked as a clerk in a town resort. Although they had not travelled into forested areas, the family reported seeing macaques close to their home. The second family included three brothers aged one, five and 11 years, all presenting on the same day. They lived on the island of Banggi, a forested area with numerous macaques. The 11 year-old boy had chronic myeloid leukaemia for which he took imatinib. His uncomplicated knowlesi malaria was unremarkable except for a notable absence of thrombocytopenia.

## Discussion

*Plasmodium knowlesi* is the most common cause of malaria admissions to Kudat District Hospital and affects all ages from young children to the elderly. Although the greater proportion of males, particularly among adults, is likely to be consistent with occupational forest or plantation exposure as a risk factor for knowlesi malaria, the wide age distribution suggests that this is not the only determinant of transmission. Furthermore, this report of two family clusters, one of which had not travelled outside Kudat town, suggests that transmission may be occurring close to or inside people’s homes. These findings differ from those reported from two studies in Sarawak, where a smaller proportion of knowlesi malaria cases occurred in children (8/121 [6.6%] in one study [4] and 9/106 [8.5%] in the other [10]), and no clustering of cases was reported despite the presence of communal longhouses in the study areas [4,10]. Epidemiological risk factors of knowlesi malaria have not been previously investigated in either of these areas and require further evaluation.

It was notable that in contrast to the age-distribution of other malaria species, 25% of all *P. knowlesi* cases occurred in adults over 50 years of age. This is consistent with a lack of past immunity to *P. knowlesi* in this age group and/or a relatively recent increase in the risk of exposure to this species in the Kudat region. It may also relate to greater forest exposure among older individuals, with farmers and plantation workers over-represented among this age group [7]. The finding that adult females with knowlesi malaria are older than adult males is consistent with a previous report [7] and requires further investigation, but may relate to less forest and/or vector exposure among younger females. The older age of female adults with *P. knowlesi* may also explain the finding from two previous studies that female patients are at greater risk of severe disease [4,6].

In Kapit, Sarawak, *An. latens* was recently identified as the primary *P. knowlesi* vector, with the mosquitoes biting both monkeys and humans, and four (0.4%) infected mosquitoes found in forest and farm locations [12]. Although 126 (11.7%) *An. latens* were found in longhouses, none were infected, suggesting transmission occurs away from homes [12]. A subsequent study found evidence that in Kapit *P. knowlesi* remains a zoonosis, based on an extremely high prevalence (87%) of *P. knowlesi* in long-tailed macaques, and sequencing of the csp gene and mtDNA showing a greater number of *P. knowlesi* genotypes per monkey infection than human infection, with genotypes common to both hosts and no genotype exclusive to either [17].

In Kudat, the wide age distribution and clustering of cases suggest that the *P. knowlesi* vector may be biting humans close to or inside people’s houses. Previously *An. balabacensis* was known to be the primary vector of human malaria in Sabah [18], with *Anopheles flavirostris* also identified as a potent *P. falciparum* vector on Banggi Island [19]. *Anopheles balabacensis* have been shown to readily bite monkeys at the canopy level [20], and a *P. knowlesi*-infected *An balabacensis* was recently found in Ranau [13]. Of significance for potential human-to-human malaria transmission, *An. balabacensis* have been shown to exhibit “learning behaviour” in relation to host preference, and may also demonstrate “habitat loyalty” by returning to previous feeding sites [21]. Furthermore, *An. balabacensis* has been shown to be a highly efficient vector [13], and is closely related to *Anopheles dirus*, the primary malaria vector in the Mekong region, which maintains high levels of malaria endemicity at low population densities [22], is capable of adapting to deforestation [23], and in Southern Vietnam was found to be positive for *P. knowlesi* DNA [24].

More recently however, *An. donaldi* was shown to have replaced *An. balabacensis* as the primary species in the Kinabatangan region of Sabah [14]. The peak outdoor biting time for this mosquito occurs from 18.00-19.00 hours, when humans are often outside their homes, and indoor biting occurs throughout the night. Although sporozoites were detected in *An. donaldi* in the Kinabatangan region [14], species identification was not performed and further studies are required to determine the role of this species in the transmission of knowlesi malaria.

A strong association was demonstrated between knowlesi malaria cases and rainfall, with a lag time of three to five months. Anopheles mosquitoes depend on rainfall to provide aquatic breeding sites, and *An. balabacensis* and *An. donaldi* have both been shown to have increased parity rates during the months of greatest rainfall [14]. An association between rainfall and falciparum and vivax malaria prevalence has been well documented [25-29], and can be used to assist with malaria early warning systems and prediction of epidemics [30,31]. This association should also be considered when planning for control of knowlesi malaria. However, alternative explanations for seasonal variation in knowlesi malaria such as other climatic variables and seasonal plantation work were not explored in this study and require further investigation.

Although the numbers of *P. vivax* and *P. falciparum* in this study were small, and could only be assessed over a three-year period, the number of *P. vivax* cases increased during the study period along with *P. knowlesi,* while a modest decrease was seen in *P. falciparum* cases. Larger prospective studies will be required to further evaluate the interaction between malaria species, which may have implications for malaria control.

This study had several limitations. First, the retrospective design did not allow the collection of information on where patients acquired their malaria infection. This prevented detailed assessment of forest exposure as a risk factor for knowlesi malaria, and may also have influenced the association between malaria cases and rainfall recorded at Kudat Meteorological Station. In particular, information was lacking on which patients came from Banggi Island, which, although only a short distance from Kudat town, may represent a different geographical setting with different transmission dynamics. Second, identification of case clusters was based only on patients with the same family name recorded in microscopy books, and this may have led to an underestimation of case clustering. Third, only a small number of patients with microscopy-diagnosed *P. falciparum* or *P. vivax* had PCR performed, and microscopy reports were therefore relied on for analyses involving these species. It is possible that some of the patients diagnosed by microscopy as *P. falciparum* or *P. vivax* had *P. knowlesi*. However, despite the potential for this to dilute age-related differences among species, a significant difference in age distribution was noted between *P. knowlesi* and microscopy-diagnosed *P .falciparum* or *P. vivax*, making the estimates of age-related differences conservative. Finally, this study involved the use, prior to June 2011, of a nested PCR assay (using Pmk8-Pmk9 primers) that has been associated with cross-reactivity with *P. vivax* DNA, and false-positive PCR findings of mixed *P. knowlesi/P. vivax* in true *P. vivax* mono-infections [32]. In this study this may account for the one third of patients diagnosed by microscopy with *P. vivax* mono-infection prior to June 2011, and subsequently found to have mixed *P. vivax/P. knowlesi* infections by PCR. Cross-reactivity with *P. vivax* isolates has not been reported with the real-time PCR assay [15], and it was notable that after the introduction of this method there were no *P. knowlesi/P. vivax* mixed infections diagnosed by PCR despite an increase in microscopy-diagnosed *P. vivax* mixed and mono-infections over this time.

Malaysia has made impressive progress in reducing malaria prevalence in recent years; however, *P. knowlesi* cases appear to be increasing, with the species now accounting for the majority of malaria admissions to Kudat Hospital as well as other district hospitals throughout Sabah and Sarawak [3,4,9]. The simian host reservoir of this zoonotic species presents particular challenges for malaria control, and will hinder the progress of malaria elimination in Malaysia. In addition, the increasing dominance of *P. knowlesi* and the possibility of transmission occurring close to or inside people’s homes may increase the possibility of a switch to the human host. Prospective studies on epidemiological risk factors, vectors and transmission dynamics of *P. knowlesi* in Sabah, including the possibility of human-to-human transmission, are required in order to develop strategies for knowlesi malaria control.

## Competing interests

The authors declare that they have no competing interests.

## Authors’ contributions

BEB, NMA, TWY, TW, PD and MJG conceived and designed the study. FA performed the PCR assays at Sabah State Reference Laboratory. BEB collected and analysed the data. BEB and NMA wrote the manuscript. All authors read and approved the final manuscript.
